# The identity group as a source of social influence for individuals with concealable stigmatized identities

**DOI:** 10.1371/journal.pone.0309687

**Published:** 2024-09-11

**Authors:** Andrew C. Cortopassi, Diane M. Quinn, Gandalf Nicolas

**Affiliations:** 1 Department of Psychology, Rutgers University – New Brunswick, Piscataway, New Jersey, United States of America; 2 Psychological Sciences Department, University of Connecticut, Storrs, Connecticut, United States of America; University of New South Wales, AUSTRALIA

## Abstract

Many people who are stigmatized along concealable features (e.g., sexual minorities or people with mental illness) anticipate social rejection due to their features and associated labels, and these beliefs are a prominent predictor of psychological distress. While ecological approaches to stigma research have highlighted the social basis of these two related outcomes, it typically has focused on the impact of non-stigmatized counterparts. Also embedded in the social environment are similarly-stigmatized others who, in concealing, may be less accessible to the individual. Given the centrality of psychological distress and rejection concerns as a relational self-conception in attachment theories, we tested if identity-based rejection sensitivity and distress may emerge from diminished access to similarly-stigmatized others as identity group members. Leveraging the University as a partially-controlled, naturalistic setting, we collected measures of concealment, identity-based rejection sensitivity, and psychological distress from undergraduate students in introductory psychology courses who reported a concealable stigmatized identity (*N* = 355; *k* = 15 identity groups). With concealment aggregated to the level of the identity group, multi-level modeling showed that concealment by similarly-stigmatized students was positively associated with both individuals’ identity-based rejection sensitivity and their psychological distress. Moreover, rejection sensitivity mediated the association of group-level concealment and distress. Findings suggest that rejection concerns and distress may emerge from identity group inaccessibility in the social environment, with the association of concerns and distress possibly contextualized by underlying group attachment dynamics. Results reveal the identity group as a novel source of social influence in the lives of individuals with concealable stigmatized identities.

## Introduction

People who are stigmatized along concealable features exhibit variation in socially-salient, internal processes—such as in sexual attraction, in thought or mood, or in past experiences—that has been labeled and negatively-stereotyped within the broader culture [1]. Because these features are less visually-salient, people are able to conceal them, and the stigmatized individual can exert agency in determining who knows about their features and thus the likelihood that they will be used by perceivers to negatively-categorize them [2]. The ability to conceal features has clear advantages for navigating social life, facilitating the evasion of social rejection for sexual minorities, people with mental illness, or people who have experienced sexual assault (e.g., [3]). Yet, many of these people nevertheless report significant preoccupation with social rejection due to their features and associated labels (e.g., [4, 5]), which is a prominent predictor of psychological distress (i.e., everyday symptoms of depression and anxiety; [6, 7]). The ability to conceal appears to come at significant psychological costs for the individual stigmatized along concealable features.

### The individual in context

**Fig 1:**
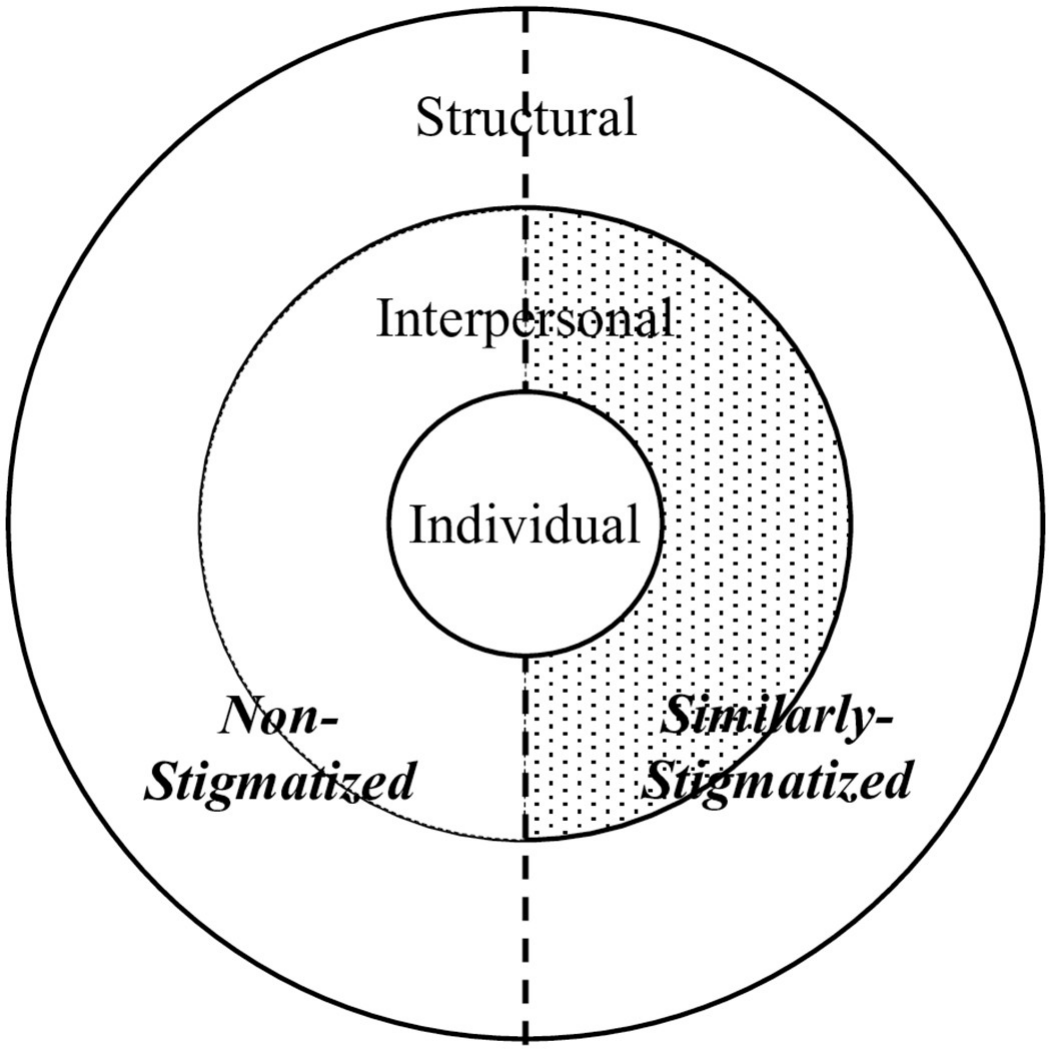
The stigmatized individual in the social environment. *Note*. The stigmatized individual situated in the social environment, modified from [12] by differentiating people’s relationship to the stigmatized individual: similarly-stigmatized or non-stigmatized counterpart. The patterned *interpersonal*:*similarly-stigmatized* region indicates the relative gap in our knowledge.

Attending to these notions in the social construction of stigma [1], ecological approaches to stigma research have shed light on this predicament. While the mental health consequences of living in hostile social environments have been a core focus of this contextual research (e.g., see [8] for a recent review for sexual minorities), research similarly has highlighted external sources of heightened rejection concerns, each reflective of hostile social environments [e.g., 9]. Often, people learn to expect rejection—typically from previous experiences with it, such as in negative disclosure reactions [10], but also vicariously through observation, such as through seeing negative media depictions [11]. Indeed, such feedback depends on the actions of others, as individuals always exist within the context of the social environment. As can be seen in Fig 1, the stigmatized individual is shaped by (but also shapes) at least two broader levels of influence. They exist among interpersonal contacts and within the context of structures that these contacts build or maintain [12]. In multi-level studies, individual expectations of rejection are predicted by negativity in the environment—captured interpersonally in stereotypes and prejudice endorsed by non-stigmatized counterparts (e.g., [6, 13]) or by proxy in structures, such as laws or policies that reflect the negative treatment of stigmatized people in a given place and time (e.g., [14]). In all, it is not surprising that both expectations of rejection and psychological distress would be elevated in social contexts where non-stigmatized others harbor negative views toward people stigmatized similarly to oneself.

Typically absent in this contextual approach, however, is the influence of *similarly-stigmatized others* on the individual. Despite being the targets of stigmatization themselves, how stigmatized individuals think, feel, and behave within the context of their own stigmatization likely contributes to the predicament of other stigmatized individuals. Although some research has examined the impact of similar others in terms of structures (e.g., effect of LGBTQ clubs in schools on mental health for sexual minorities; [15]), research has not examined their impact at the *interpersonal* level. We propose that the social environment can be differentiated based on people’s relationship to the stigmatized individual (i.e., non-stigmatized counterpart versus similarly-stigmatized), and that heightened rejection concerns and psychological distress can reflect the influence of similarly-stigmatized others at the interpersonal level. One way that these related outcomes may arise is that, when individuals conceal (i.e., make an active effort to keep their features, labels, or otherwise identities hidden [16]), they may be concealing from similarly-stigmatized others.

Sometimes when individuals conceal, they have identified specific targets: non-stigmatized counterparts who hold (or they anticipate holding) stigmatizing views (i.e., disclosing selectively; [17]). However, people are not always cognizant from whom they conceal (i.e., whether they are non-stigmatized or similarly-stigmatized). Stigmatized or not, the features—such as sexual attraction or mood—often can be the same across people: internal and thus concealable. The difference is that some variation in those features, such as same-gender attraction or low mood, is particularly salient, labeled, and negatively-stereotyped [1]. Thus, in many concealment situations, the “identity” of the other person in the interaction is ambiguous to the stigmatized individual. In other situations, expressed stigmatizing views may be a cue and make the non-stigmatized person known to the stigmatized individual, but there also may be other people present in these interactions. Again, these bystanders may be similarly-stigmatized, but because the features are concealable, the individual may not recognize them as such, especially if these bystanders are themselves concealing. In both of these cases, whether unwitting or collateral, similarly-stigmatized individuals can be in the radius of an individual’s concealment. It is also important to note, however, that concealment from (suspected) similar others might proceed intentionally, if the individual does not wish to be associated with them.

Whatever the situational circumstances surrounding an individual’s decision to conceal, concealment is likely to affect similarly-stigmatized others. While some theorizing has suggested the impact of concealment on stigma reduction among non-stigmatized counterparts [18], another possibility is that concealment keeps similarly-stigmatized individuals “apart” from one another as *identity group members*. Indeed, being stigmatized is theorized to change the self, as people construct meaning around their stigmatized features and their associated labels, denoting concealable stigmatized *identities* [6]. Yet, for many people, the development or maintenance of a positive identity can be a challenge [10]. In the aggregate, when many individuals conceal, the group as a whole is more concealed, and one possibility for heightened rejection concerns and increased psychological distress is that they emerge from individuals’ diminished contact with group members in the social environment. Anxious expectations of social rejection may represent a *negative identity self-conception* emergent from (or amplified in the context of) diminished access to similarly-stigmatized others. It may be that this underlying group-self dynamic, in turn, facilitates psychological distress for individuals with concealable stigmatized identities.

### Identity-based rejection sensitivity

The idea that rejection concerns could indicate an underlying identity construct, derived in relation to other people, coheres with classic conceptualizations of *rejection sensitivity*. In personality psychology, rejection sensitivity is theorized as the manifestation of attachment anxiety [19], which is a facet of self [20]. People high in rejection sensitivity not only are worried about rejection, but they also perceive it in ambiguous situations and often have intense emotional reactions to it [19]. Rejection sensitivity is a function of two appraisals: beliefs in the likelihood of rejection (cognitive component) and concerns that it will happen (affective component). The affective component is theorized to interact with the cognitive to produce the rejection sensitivity dynamic: a cross-situational, trait-like *disposition* to expect rejection. This dynamic is in line with conceptualizations of attachment anxiety [20], which represents generalized relationship insecurity rooted in a negative self-conception. While stigma research on rejection concerns sometimes has adopted the measurement of various forms of *stigma-related* rejection sensitivity [e.g., 4, 5], examining the impact of rejection concerns due to a feature, label, or otherwise identity, typically this research has not explored the broader theoretical implications of the construct: its basis in attachment anxiety.

Attachment anxiety is characterized by increased vigilance for information that confirms negative beliefs about the self in relationships [20]. Corresponding to perceptions of the self as unworthy of love, care, and acceptance, attachment anxiety is a consistent predictor of psychological distress [21]. Although attachment processes are typically conceptualized as intra-individual constructs (e.g., attachment styles; [22]), attachment is an *interpersonal* phenomenon. Positive attachment relationships are what provide a secure base for people to explore the world with reduced rejection concerns [23, 24], inspiring confidence in the self as worthy of love, care, and acceptance from others [25]. Classically, the secure base relationship is the childhood bond with primary caregivers [23, 24, 26], but attachment dynamics also have been shown to operate at the group-level [27, 28], and recent theorizing has posited that stigmatized groups may play a secure base role for stigmatized individuals navigating intergroup contexts [29]. Thus, in line with its basis in attachment theory [19], *identity-based* rejection sensitivity may emerge from thwarted group attachment dynamics. We note that, although people are likely sensitive to rejection based on features and their labels, we use “identity-based rejection sensitivity” in this article to highlight the construct’s basis in self-perception. For individuals with concealable stigmatized identities, concealment likely undermines secure attachment relationships among group members, and without the group, individuals may have fewer resources for building the confidence to navigate the world with attenuated rejection concerns.

### Current research

In the current research, we synthesize these ideas to examine the identity group as an external source of rejection concerns and psychological distress for individuals with concealable stigmatized identities. We propose that these concerns can represent identity-based rejection sensitivity, reflecting a negative identity self-conception derived through diminished access to similarly-stigmatized others as group members. Although it is individuals who conceal, this individual-level behavior can have social consequences in the aggregate, such that the group in the social environment is more concealed. In the collective, concealment may keep proximate, similarly-stigmatized people “apart” from one another, and these higher-level, group processes may shape the self to impact psychological well-being. Ultimately, a broadened conception of the social environment to include similarly-stigmatized others could provide greater insight into the origin (or maintenance) of heightened rejection concerns and their prediction of psychological distress for individuals with concealable stigmatized identities. Here, we hypothesized that concealment by identity group members would contextualize the association of identity-based rejection sensitivity and psychological distress for individuals.

## Methods

### Recruitment and enrollment

We collected measures of active concealment, identity-based rejection sensitivity, and psychological distress from students in the psychology participant pool at the University of Connecticut in the Spring 2022 semester. Every semester, the psychology department offers a handful of introductory psychology classes, and all students are required to complete several research studies for partial course credit. Because they are classmates, participants recruited from the participant pool plausibly could interact with and know one another (but perhaps not one another’s identities). The participant pool then can provide a mechanism to conduct a partially-controlled, naturalistic study of how individuals impact one another, with the group-level dynamics that emerge in these datasets meaningfully reflecting participants’ broader social environment. While the degree to which the broader University is self-contained is likely to dictate the utility of this recruitment strategy, the University of Connecticut is located in a very rural college town (~17,000 residents) and requires all first-year students to live on campus.

To examine our group-level hypotheses, it was necessary to maximize the number of groups in the sample. In multi-level modeling, determining sample size is complex. One review paper suggests that at least 15 groups with 5–30 observations each can suffice for unbiased fixed effects [30]. At the beginning of each semester, a few weeks before research studies become available in the online participant pool interface, all students complete an online pre-screening survey during class (i.e., mass testing). Researchers can opt to include various questions in this survey to suit their research needs (i.e., measure inclusion or exclusion criteria). To inform our recruitment efforts, we included several questions in order to estimate the prevalence of 16 concealable stigmatized identities in the entire participant pool population. We used these data to devise a ranked-ordered priority list for our subsequent study recruitment (see S1 Appendix). For each of our 16 identity groups (see Table 1), we aimed to enroll 20 participants per group, but given norms in the literature [30], we retained groups with at least five participants.

**Table 1:** Sample composition and concealment dynamics in the social environment (N = 355).

	Sample	Active
	Prevalence	Concealment
**Identity Group**	*n* (%)	*M* (*SD*)
Sexual Assault	30 (8.5)	2.0 (0.6)
Childhood Emotional Abuse	29 (8.2)	2.2 (0.8)
Nicotine Addiction	27 (7.6)	1.4 (0.3)
ADHD	27 (7.6)	1.6 (0.6)
Bisexual	27 (7.6)	1.5 (0.7)
OCD	26 (7.3)	1.6 (0.4)
Anxiety Disorder	25 (7.0)	2.2 (0.7)
Asthma	24 (6.8)	1.2 (0.2)
Self-Injury	24 (6.8)	2.5 (0.9)
Inflammatory Bowel Disease	23 (6.5)	1.6 (0.8)
Pornography Addiction	23 (6.5)	2.2 (0.8)
Poverty	22 (6.2)	1.8 (0.7)
Major Depression	18 (5.1)	2.5 (0.8)
Anorexia Nervosa	17 (4.8)	2.0 (0.9)
Drug Dependence	13 (3.7)	2.1 (0.8)

Once the online participant pool interface became active, our study was made visible to all students in the participant pool, listed among all others offered that semester. Individuals who signed up for our study read an information sheet and first provided informed consent to complete an anonymous, online identity screening checklist to determine their eligibility. This checklist included our targeted identities, as well as several “filler” identities. If participant endorsed one of our targeted identities, they were eligible to participate in our study and answered questions about that identity in the main survey. In order to ensure our recruitment goal, any participant who endorsed multiple targeted identities on our screening checklist was assigned a group. In these instances, participants answered questions for whichever of their endorsed identities ranked highest on our recruitment priority list (i.e., least prevalent among students in the participant pool).

Among the students who completed the screening checklist, 554 individuals were preliminarily-eligible and elected to participate in the main survey. Of these participants, 413 confirmed their endorsed identity from the screening checklist, answering in the affirmative to a direct question about identity possession. Other than this question, participants were not given any feedback on which responses on the checklist rendered them eligible to participate. They were simply asked to confirm the singular targeted identity they endorsed (or that was assigned). A total of 396 individuals completed the survey. Thirteen participants participated twice, so we retained the first submission. Of 383 unique participants, 365 correctly answered two item-level attention checks (We excluded these 18 participants, but results remain the same in robustness analyses; see S1 Appendix). We also excluded seven participants with missing data for concealment, rejection sensitivity, or distress. Finally, we did not recruit at least five participants for a final group (bulimia nervosa; *n* = 3) and excluded these participants.

The analytic sample consisted of 355 participants. Using *simr* [31] to simulate our data structure and a small-to-medium effect size (10,000 simulations; β = 0.2), we had 94.60% CI [94.14, 95.03] power to detect the association between group-level concealment and psychological distress. Most participants identified as White (74.6%), followed by Asian (9.0%), Black (8.7%), Multiracial (4.2%), and Native American or Hawaiian (0.9%). The minority (15.5%) identified as Hispanic. Most participants (67.6%) identified as women, while the remaining participants were male-identified (30.7%), with some genderqueer (0.6%) individuals and people identifying with multiple genders (0.9%). Of note, these values reflect valid percentages, as nine participants had missing sociodemographic data for one or more variables. Participants reported an average 19.0 (*SD* = 1.4) years of age. Finally, most participants were completely embedded in the University ecosystem, with 90.3% of participants reporting living on-campus. The remaining students lived off-campus with University peers (5.9%) or off-campus with others (3.9%). The study was not pre-registered. Data, code, and materials are available at https://osf.io/kr654/.

### Procedure

On the main study landing page, participants provided informed consent to complete an anonymous, online survey regarding their identity and well-being. They read an information sheet and decided whether to proceed. In the measures, wherever an identity or group was mentioned in a question stem, the participant’s specific identity or identity group was inserted. Measures were administered in the order described below. After the 15-minute survey, participants were debriefed, provided mental health referrals, and allocated partial course credit. The Institutional Review Board at the University of Connecticut approved all study materials and procedures.

### Measures

#### Active concealment

To measure active concealment, we administered the Quinn Active Concealment Scale [16]. This 15-item measure assesses the frequency with which people employ various strategies to conceal and/or exert energy to keep their stigmatized features, labels, or otherwise identities hidden from others. Participants responded on a scale from 1 (*never*) to 5 (*always*). The scale midpoint was labeled *half of the time*, flanked between *sometimes* and *most of the time*. Example items included: “To keep {identity} hidden from people, I use vague language when talking about my personal life,” “I try to act just the opposite of the ways that {identity group} are ‘supposed’ to act,” and “If the topic of conversation is about {identity group}, I just keep quiet and say nothing.” Critically, each item referred to concealment behavior at the University and in the company of others at the University, which contextualized the concealment behavior of identity group members in the immediate, social environment. Responses were averaged (α = .91).

#### Identity-based rejection sensitivity

We adapted the six social rejection items from the anticipated stigma scale [6]. This scale assesses people’s beliefs in the likelihood of social rejection and stigmatization-devaluation if others knew their identity. We also collected items for the latter factor (see S1 Appendix for analyses with this variable). Because we were interested in tapping a trait-like disposition to anxiously expect rejection due to an identity, we asked participants to consider the six rejection situations in general. We modeled items after the original rejection sensitivity measure [19] and its extensions to stigmatized identities (e.g., [4, 5]). For each situation, participants first rated their concern (“In general, how concerned are you that people would not want…because of [identity]?”) and then their perceptions of its likelihood (“In general, how likely is it that people would not want …because of [identity]?”). The specific situations appeared in the ellipsis.

An example situation was “to study or work with you.” This was the one modification we made from the anticipated stigma scale, as “friends stop hanging out with you” hinged specifically on others discovering the identity. For both concern and likelihood, responses were measured on 6-point bipolar scales, ranging from 1 (*very unconcerned/unlikely*) to 6 (*very concerned/likely*). The bivariate association between averages for concern (α = .93) and likelihood (α = .92) variables was high, β = .81, *p* < .001. As in the classic operationalization [19], concern and likelihood ratings for each situation were multiplied. These six concern-likelihood products (α = .93) were averaged to give an identity-based rejection sensitivity score.

#### Psychological distress

As in previous research with concealable stigmatized identities [6, 7], we operationalized psychological distress with symptoms of depression and anxiety and administered the widely-used Center for Epidemiological Studies–Depression (CES-D; [32]) and the Spielberger Trait Anxiety Inventory (STAI; [33]) scales. For the 20 items of the CES-D, participants indicated the frequency with which they experienced a variety of symptoms in the past two weeks on a scale from 0 (*rarely or none of the time*) to 3 (*most or all of the time*). With the STAI, participants indicated the frequency of 20 symptoms in general, with frequency ranging from 1 (*almost never*) to 4 (*almost always*). While often used separately to screen for potential anxiety or depressive disorders, there is significant overlap in the scales’ contents. Thus, we did not focus on discriminant prediction of the CES-D (α = .92) and the STAI (α = .93), which were highly associated, β = .81, p < .001. Rather, we took the sum of standardized values for all items across the scales for a psychological distress composite (α = .96).

### Additional variables

We included a few exploratory variables alongside our measures of interest. See S1 Appendix.

### Analyses

Analyses were conducted in a few steps. We first obtained descriptive statistics and bivariate associations between study variables. Bivariate associations are reported as standardized regression coefficients from mixed regression models, because participants were clustered within groups. Then, we prepared a dataset to describe the concealment dynamics in the social environment and to examine its proposed contextualizing role. In the absence of best practices for quantifying the confounding effect of a variable (i.e., contextualizing the relationship between two variables with a third), we examined a mediation model, as confounding and mediation are statistically equivalent [34], with both highlighting the interrelationship among three variables. To test this prediction, we conducted a 2-1-1 mediation model [35], examining the predictive power of concealment (Level 2) on levels of identity-based rejection sensitivity (Level 1) and psychological distress (Level 1) outcomes. To show that rejection sensitivity mediates a potential relationship between group-level concealment and distress would suggest that the association between sensitivity and distress may be contextualized by (and emerge from) concealment dynamics in the social environment.

#### Modeling the influence of the identity group in the social environment

To examine the influence of group members in the social environment on the stigmatized individual, we prepared a multi-level dataset. First, we quantified the concealment dynamics of the social environment. To do so, we calculated the average concealment score for each of the 15 identity groups in the sample. Each individual participant was then assigned the derived group-level concealment score for their identity group. This group-level variable—shared among all members in an identity group in the sample—estimated the extent to which identity group members in the University environment (or at minimum, the social environment of their introductory psychology course) actively concealed. Greater levels of group-level active concealment were taken to indicate less accessibility of group members in the participants’ social environment. These values are displayed in Table 1.

Next, we derived the within-group component of our concealment variable. This partitioning of the concealment variable accommodates the structure of the data [36] and permits locating *where* an association occurs: aggregate concealment scores capture the between-group component, but the within-group effect is estimated at the individual-level by subtracting the identity group average from the concealment score for each individual participant in that group. By defining each individual’s score relative to their group, *group-mean-centering* means that individuals’ concealment scores are compared only to other group members, accounting for the specific features, label, or otherwise identity concealed. In simultaneous regression models, components control for one another, and the magnitude of the between- and within-group effects can meaningfully be compared [36]. This comparison tests whether the behavior of the group is a strong, independent facilitator of the association between identity-based rejection sensitivity and psychological distress—above and beyond the influence at the individual-level. Thus, all regression models included both between-group and within-group concealment components.

#### Multi-level mediation analysis

We examined the proposed 2-1-1 mediation model following the four-step, regression-based approach to mediation [37]. First, group-level concealment was examined as a predictor of psychological distress (total effect). Second, group-level concealment was examined as a predictor of identity-based rejection sensitivity (path *a*). Third, rejection sensitivity was examined as a predictor of distress (path *b*), controlling for group-level concealment (direct effect). Finally, merely to highlight the interrelationship of the three variables, we used the *mediation* package [38] in R to calculate the indirect effect of group-level concealment on distress through rejection sensitivity (parameter *ab*) using 5,000 simulations. A significant mediation would support the contextual role of group-level concealment in the association of sensitivity and distress. All analyses controlled for group-mean-centered concealment, and to account for the non-independence of group members, mixed regression models were used with the *lme4* [39] and *lmerTest* [40] R packages, specifying the identity group as a random intercept.

## Results

### Sample characteristics

Bivariate associations among variables and descriptive information are provided in Table 2. In this sample, psychological distress was high. Based on diagnostic thresholds for mild-to-moderate depression and anxiety, two-thirds of participants (65.9%) met criteria for depression (*M* = 21.9, *SD* = 12.1), and four-fifths (80.0%) for anxiety (*M* = 49.7, *SD* = 11.4). The range in active concealment is displayed in Table 1. Values varied by identity group. Participants were not extremely sensitive to identity-based rejection, as the average situational concern-likelihood product placed participants in the first quartile of possible rejection sensitivity scores (possible range 1–36). Cross-situational concern (*M* = 2.7, *SD* = 1.4) and likelihood (*M* = 2.5, *SD* = 1.2) ratings were similar.

**Table 2:** Bivariate associations and descriptive information.

	1	2	3	M (*SD*)
1. Group-Level Concealment	-			1.9 (0.4)
2. Rejection Sensitivity	.36	-		8.7 (7.5)
3. Psychological Distress	.34	.40	-	0.0 (24.8)

Note. All *p*s < .001

### Multi-level mediation

Results from mixed regression models testing the contextual role of group-level concealment are presented in Table 3. Results supported hypotheses. Identity-based rejection sensitivity was associated with increased psychological distress, and group-level concealment was positively associated with both variables, suggesting its contextualizing role.

**Table 3:** Results from mixed models testing the contextual role of group-level concealment.

	Identity-Based			Psychological		
	Rejection Sensitivity			Distress		
	*B*	*SE*	*p*	*B*	*SE*	*p*
Group-Level Concealment	6.95	1.15	< .001	14.40	3.37	< .001
GMC Concealment	5.42	0.48	< .001	5.48	2.02	.007
Identity-Based Rejection Sensitivity	-	-	-	1.00	0.19	< .001
*Total Effect*:						
Group-Level Concealment	-	-	-	21.31	3.48	< .001
GMC Concealment	-	-	-	10.88	1.80	< .001
*Indirect Effect*:				*ab*	95% CI	
Group-Level through Rejection Sensitivity				6.93	3.76, 10.61	

Note. GMC = Group-Mean-Centered

**Fig 2:**
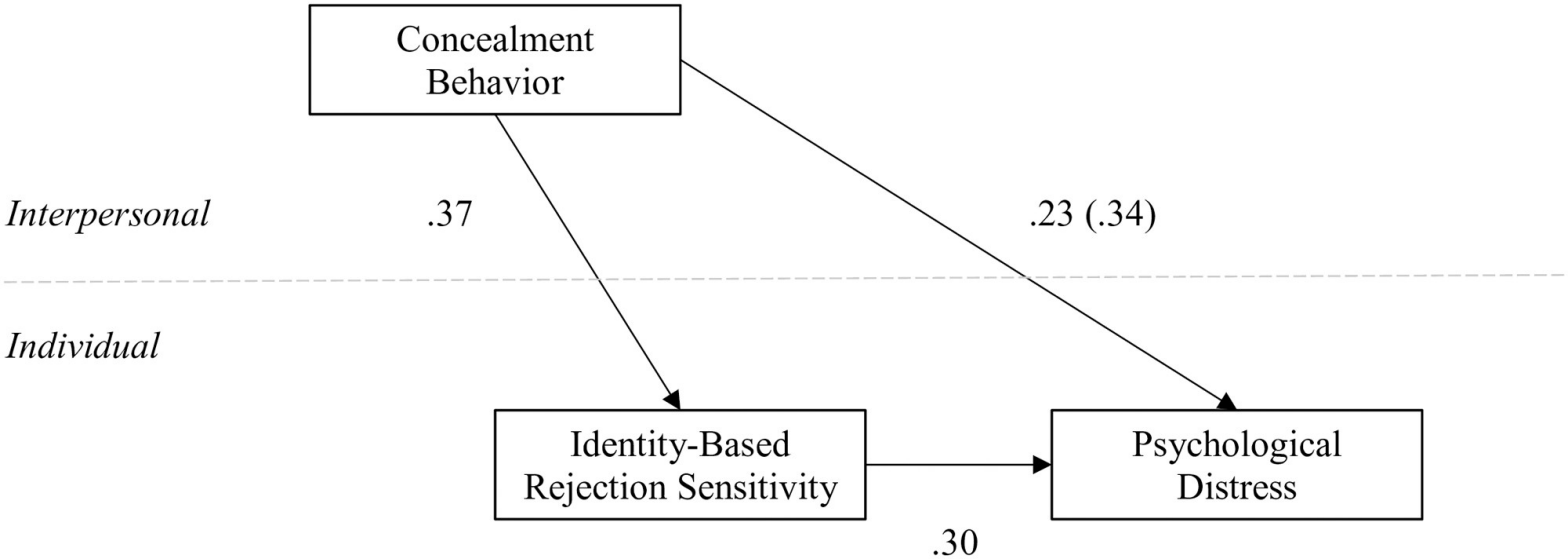
Impact of concealment by similarly-stigmatized others in the social environment. Note. All *p*s < .001; Total effect in parentheses.

This contextual role was further supported by simulations for the indirect effect, which showed that rejection sensitivity mediated 32.3% of the association between group-level concealment and distress. Notably, individual-level concealment (i.e., group-mean-centered concealment) was a weaker predictor of both rejection sensitivity and distress than at the group-level. For example, group-level concealment was associated with distress at a magnitude almost two to three times greater, depending on the path tested. Results are depicted with standardized regression coefficients in Fig 2.

## Discussion

Even though their identities are based on labeled features that are less visually-salient, people with concealable stigmatized identities report significant concerns about identity-based rejection [6], and these concerns are a prominent predictor of psychological distress [7]. Drawing from attachment theory, we proposed that these anxious beliefs partially persist because they reflect a negative self-conception based in group attachment dynamics. While concealment can prevent social rejection, it may also inhibit the ability to form secure attachment relationships with *identity group members*, exacerbating rejection sensitivity and undermining mental health. In the current research, we found that the association of identity-based rejection sensitivity and psychological distress could be partially traced to concealment by one’s group members in the social environment. Our data uncover an additional facet to the predicament of living with a concealable stigmatized identity [6]. With less access to group members, people may be even less confident to navigate the world with their stigmatized identities and experience diminished mental health.

A prominent finding from our data is that concealment at both the group- and individual-level predicted outcomes of rejection sensitivity and psychological distress, controlling for the other. It would stand to reason that individual-level concealment could contextualize the association of sensitivity and distress: as individuals conceal from (presumably) non-stigmatized individuals, they encounter fewer opportunities to gather evidence that people are not as rejecting as they anticipate [41], as well as present fewer opportunities to be identified by group members. Yet, the mechanism appears strongly to implicate *other group members*, as group-level concealment outperformed the individual-level component in predicting both outcomes. Rather than the individual’s own actions, it is the behavior of the group, as a whole, that more strongly contextualizes the association of sensitivity and distress for individual group members. Although stigma research typically locates processes and outcomes in individuals [12], this finding partially absolves the individual, unveiling the strong role of the group in shaping individual-level outcomes. The irony, however, is that while individual-level concealment contributes less than at the level of the group, it still is individuals whose concealment behavior aggregates at the group-level.

As our group-level results contextualize an association central in attachment theory (e.g., [21]), they prompt questions for future theoretical work. Namely, does group-level concealment undermine the *quantity* or *quality* of group relationships? Classically, attachment processes are considered to reflect the quality of existing relationships [23, 24, 26]. Greater concealment may be indicative of existing relationships with group members who provide unreliable, inconsistent access to positive relationships. Those who conceal may aim to shield themselves from rejection from non-stigmatized counterparts, but they may also aim to distance themselves from group members, and such behavior could undermine secure group attachment. Indeed, not only does identity-based rejection sensitivity prominently predict distress for sexual minority men [4], but recent research highlights that their group relationships can be distressing, as sexual minority men often compete in a variety of ways to distinguish and distance themselves from one another [42]. The fact that strained group relationships and rejection sensitivity both predict distress for many sexual minority men mirrors the three-variable, group-level process (i.e., group concealment) shown in the current research, possibly suggesting underlying diminished secure (group) relationships for these men.

However, a strict quality argument depends on existing group attachment relationships. Concealment likely undermines the establishment of these relationships by impacting both the quantity and quality of relationships. The more that group members in the environment conceal, the fewer opportunities to access potential attachment figures—the quantity of relationships. However, even in the absence of concealment, a larger pool of identifiable, potential attachment figures does not necessarily mean that those group relationships would yield a secure group attachment for a particular individual. Like the formation of any close relationship, multiple personality and individual factors contribute to this outcome for two people with the same identity. However, with more identifiable, potential attachment figures in the environment, the greater the likelihood that one or more group member relationships will foster secure group attachment. Concealment then likely reduces the conditional probability of encountering a group member who could foster secure group attachment. Although attachment at the group level [27, 28] and its secure base capacity [29] have been documented, future research should examine explicitly if attachment dynamics are at play in the associations shown in the current research and how concealable stigmatized identity group attachment diverges from classic relationship contexts.

### Limitations

The current research does have some limitations. These data are cross-sectional, precluding conclusions about directions between variables. We propose that, in the context of group members’ concealment, sensitivity is the predictor of distress, but distress may also predict sensitivity: cognitive biases in depression and anxiety can shape people’s anticipation of relational threat [43, 44]. It is also possible that distress among individuals predicts concealment, which may then be reflected in group-level averages of concealment in the social environment. People vary in their tendency to conceal distress [45], which may extend to their features, labels, or otherwise identities due to concerns of being “outed.” Finally, although our use of multi-level mediation analysis was meant simply to test for a significant interrelationship among all three variables, there are noted limitations to mediation analysis as a tool for examining third-variable relationships, in addition to their limitations in determining causality [46]. Future research may wish to employ additional methods (e.g., longitudinal designs or different approaches to contextual analysis) to increase confidence in directions and relationships among the variables explored in these studies.

There are also constraints on generalizability. The process seen here may depend on the developmental characteristics specific to our sample. Undergraduate matriculation typically occurs at the end of adolescence [47]. Due to their age, participants may not have been living with their identities for long and thus may have yet to establish many group relationships, making it a high priority for them. Similarly, college marks a significant developmental milestone, as people assume newfound independence apart from childhood origins [47]. Even if they have developed group relationships, the thrust into the undergraduate environment can be a significant stressor, as people often must update relationships and support systems with new relationships. In contrast, participants in a community sample would likely be much older and would likely not be in the midst of a significant transition, and patterns among the variables in the current research may be less pronounced. Thus, although the sampling of undergraduate psychology students allowed a naturalistic, partially-controlled test of our hypothesis, the process observed here may be an artefact of participants’ developmental circumstances and the study setting. Future research should evaluate the generalizability of the current findings.

## Conclusions

This research is some of the first to consider the role of the identity group in shaping the predicament of living with a concealable stigmatized identity. Our findings provide some context for the association of identity-based rejection sensitivity and psychological distress, suggesting that it may partially reflect (thwarted) attachment relationships with group members. With some exceptions, mainly studies measuring structural indications of prejudicial environments facilitated by *non-stigmatized counterparts* (see [48]), stigma research often does not situate the individual within the broader social context. Our research not only situates the individual in the broader social environment but locates these external influences more proximally than structures and considers the opposite perspective, examining how *similarly-stigmatized others* in the social environment—the identity group context—shape the individual. Our aim in the current research was to broaden and diversify the scope of stigma research. In leveraging a social-developmental, multi-level framework, we hoped to advance our understanding of the predicament of living with a concealable stigmatized identity [6].

## Supporting information

S1 Appendix (DOCX)
