# Supplementary material for: The identity group as a source of social influence for individuals with concealable stigmatized identities
Source: PLoS One. 2024 Sep 11;19(9):e0309687. doi: 10.1371/journal.pone.0309687 (PMC11389917; doi:10.1371/journal.pone.0309687)
Supplement: S1 Appendix — (DOCX) [file pone.0309687.s001.docx]

**Appendix to Supplement**

- Measures List
- Recruitment Details
- Robustness Analyses
  - Removing Groups (Sensitivity)
  - Attention Check Misses
- Supplementary & Sensitivity Analyses

**Full Measures List**

|  |  |
| --- | --- |
|  |  |
| Background | Self in University |
|  |  |
|  | Outness |
|  | Active Concealment |
|  |  |
| Identity | Rejection Sensitivity |
|  | Centrality |
|  | Salience |
|  |  |
| Mental Health | CES-D |
|  | STAI |
|  |  |

**Recruitment**

This table presents the total number of people reporting each of our targeted identities during the mass pre-testing survey conducted at the beginning of the semester (prior to our recruitment). Identity totals are not exclusive of others. The assigned rank indicates priority. If participants reported multiple identities, they were assigned to answer questions for whichever ranked highest (i.e., least prevalent).

|  |  |  |
| --- | --- | --- |
| **Identity Group** | **Estimated**  **Total** | **Assigned**  **Rank** |
| Sexual Assault | 86 | 9 |
| Childhood Emotional Abuse | 68 | 5 |
| Nicotine Addiction | 44 | 2 |
| ADHD | 75 | 8 |
| Bisexual | 141 | 13 |
| OCD | 72 | 6 |
| Anxiety Disorder | 360 | 1 |
| Asthma | 224 | 14 |
| Self-Injury | 73 | 7 |
| Inflammatory Bowel Disease | 42 | 1 |
| Pornography Addiction | 54 | 4 |
| Poverty | 91 | 10 |
| Major Depression | 230 | 15 |
| Anorexia Nervosa | 121 | 12 |
| Drug Dependence | 48 | 3 |
| Bulimia Nervosa | 121 | 11 |

Although asthma is commonly considered in research on concealable stigmatized chronic physical illnesses, there is question whether it qualifies as a stigmatized identity. We chose to recruit people with asthma, as there is research to suggest that it is stigmatized, particularly among kids and adolescents. However, we conducted analyses without these participants to examine the robustness of our results (see next section).

**Robustness Analyses:**

**Removal of Asthma Group**

**(N = 331)**

To examine the robustness of our results, we conduct our analyses without the asthma group. The conclusions we make form results remain the same. These results are presented in the table below.

|  |  |  |  |  |  |  |  |
| --- | --- | --- | --- | --- | --- | --- | --- |
|  | Identity-Based | | |  | Psychological | | |
|  | Rejection Sensitivity | | |  | Distress | | |
|  | *B* | *SE* | *p* |  | *B* | *SE* | *p* |
| Group-Level Concealment | 5.87 | 1.21 | <.001 |  | 13.29 | 3.81 | .004 |
| Group-Mean-Centered Concealment | 5.46 | 0.50 | <.001 |  | 5.80 | 2.03 | .004 |
| Identity-Based Rejection Sensitivity | - | - | - |  | 0.98 | 0.19 | <.001 |
|  |  |  |  |  |  |  |  |
| *Total Effect*: |  |  |  |  |  |  |  |
| Group-Level Concealment | - | - | - |  | 18.98 | 3.91 | <.001 |
| Group-Mean-Centered Concealment | - | - | - |  | 11.13 | 1.80 | <.001 |
|  |  |  |  |  |  |  |  |
| *Indirect Effect*: |  |  |  |  | *ab* | 95% CI | |
| Group-Level through Rejection Sensitivity |  |  |  |  | 5.73 | 2.82, 9.18 | |

**Robustness Analyses:**

**Removal of Depression and Anxiety Groups**

**(N = 312)**

Because our psychological distress composite is comprised of anxiety and depression symptoms, it is possible that these identities confound results. To examine the robustness of our results, we conduct our analyses without the depression and anxiety groups. The conclusions we make form results remain the same. These results are presented in the table below.

|  |  |  |  |  |  |  |  |
| --- | --- | --- | --- | --- | --- | --- | --- |
|  | Identity-Based | | |  | Psychological | | |
|  | Rejection Sensitivity | | |  | Distress | | |
|  | *B* | *SE* | *p* |  | *B* | *SE* | *p* |
| Group-Level Concealment | 6.43 | 1.37 | <.001 |  | 13.86 | 3.83 | .003 |
| Group-Mean-Centered Concealment | 5.15 | 0.51 | <.001 |  | 4.93 | 2.24 | .028 |
| Identity-Based Rejection Sensitivity | - | - | - |  | 0.97 | 0.21 | <.001 |
|  |  |  |  |  |  |  |  |
| *Total Effect*: |  |  |  |  |  |  |  |
| Group-Level Concealment | - | - | - |  | 19.95 | 3.92 | <.001 |
| Group-Mean-Centered Concealment | - | - | - |  | 9.95 | 2.00 | <.001 |
|  |  |  |  |  |  |  |  |
| *Indirect Effect*: |  |  |  |  | *ab* | 95% CI | |
| Group-Level through Rejection Sensitivity |  |  |  |  | 6.14 | 2.81, 10.20 | |

**Robustness Analyses:**

**Removal of Attention Check Misses**

**(N = 371)**

To examine the robustness of our results, we conduct our analyses with participants who missed attention check questions. The conclusions we make form results remain the same. These results are presented in the table below.

|  |  |  |  |  |  |  |  |
| --- | --- | --- | --- | --- | --- | --- | --- |
|  | Identity-Based | | |  | Psychological | | |
|  | Rejection Sensitivity | | |  | Distress | | |
|  | *B* | *SE* | *p* |  | *B* | *SE* | *p* |
| Group-Level Concealment | 6.75 | 1.18 | <.001 |  | 14.00 | 3.43 | <.001 |
| Group-Mean-Centered Concealment | 5.35 | 0.46 | <.001 |  | 5.60 | 1.93 | .004 |
| Identity-Based Rejection Sensitivity | - | - | - |  | 0.97 | 0.19 | <.001 |
|  |  |  |  |  |  |  |  |
| *Total Effect*: |  |  |  |  |  |  |  |
| Group-Level Concealment | - | - | - |  | 20.56 | 3.56 | <.001 |
| Group-Mean-Centered Concealment | - | - | - |  | 10.81 | 1.71 | <.001 |
|  |  |  |  |  |  |  |  |
| *Indirect Effect*: |  |  |  |  | *ab* | 95% CI | |
| Group-Level through Rejection Sensitivity |  |  |  |  | 6.58 | 3.49, 10.17 | |

**Sensitivity Analyses:**

**Model with Outness**

We also collected an outness variable, which is conceptually related to active concealment. We examined our model using outness at the group-level. The model does not reproduce with this variable. This finding highlights the specificity of active concealment in driving the group phenomenon we identified in the current work.

|  |  |  |  |  | |  | |  |  |
| --- | --- | --- | --- | --- | --- | --- | --- | --- | --- |
|  | Identity-Based | | | |  | | Psychological | | |
|  | Rejection Sensitivity | | | |  | | Distress | | |
|  | *B* | *SE* | *p* |  | | *B* | | *SE* | *p* |
| Group-Level Outness | -2.20 | 1.36 | .132 |  | | -5.05 | | 2.65 | .083 |
| Group-Mean-Centered Outness | -0.28 | 0.36 | .430 |  | | -0.20 | | 1.11 | .858 |
| Identity-Based Rejection Sensitivity | - | - | - |  | | 1.36 | | 0.16 | <.001 |
|  |  |  |  |  | |  | |  |  |
| *Total Effect*: |  |  |  |  | |  | |  |  |
| Group-Level Outness | - | - | - |  | | -8.11 | | 3.96 | .063 |
| Group-Mean-Centered Outness | - | - | - |  | | -0.58 | | 1.20 | .627 |
|  |  |  |  |  | |  | |  |  |
| *Indirect Effect*: |  |  |  |  | | *ab* | | 95% CI | |
| Group-Level through Rejection Sensitivity |  |  |  |  | | -2.96 | | -6.77, 0.77 | |

**Sensitivity Analyses:**

**Concealment versus Outness**

We also examined the impact of outness and concealment at the group-level when variables controlled for one another. Results again suggest the specificity of active concealment in the group phenomenon we present in the manuscript.

|  |  |  |  |  |  |  |  |
| --- | --- | --- | --- | --- | --- | --- | --- |
|  | Identity-Based | | |  | Psychological | | |
|  | Rejection Sensitivity | | |  | Distress | | |
|  | *B* | *SE* | *p* |  | *B* | *SE* | *p* |
| Group-Level Concealment | 9.01 | 1.45 | <.001 |  | 25.34 | 4.86 | <.001 |
| Group-Level Outness | 1.99 | 0.94 | .056 |  | 3.77 | 3.15 .253 | |

**Sensitivity Analyses:**

**Both Factors of Anticipated Stigma Scale**

We collected the other 9 items from the anticipated stigma scale (i.e., stigmatization-devaluation items). Our primary concern in these analyses was examining the discriminant association of group-level concealment with rejection sensitivity (rather than stigmatization sensitivity). In Table 4, we report full descriptive information and bivariate associations (from simple mixed regression models, accounting for the identity group) among all 4 variables. Analyses were conducted with the *lme4* and *lmerTest* packages in R.

**Supplemental Table 4**.

Bivariate Associations

| Variable | 1 | 2 | 3 | 4 | M (SD) |
| --- | --- | --- | --- | --- | --- |
| 1. Group-Level Concealment | - |  |  |  | 1.9 (0.4) |
| 2. Identity-Based Rejection Sensitivity | .36*** | - |  |  | 8.7 (7.5) |
| 3. Stigmatization Sensitivity | .21* | .75*** | - |  | 6.7 (5.4) |
| 4. Psychological Distress | .34*** | .40*** | .36*** | - | 0.0 (24.8) |

Note. * p <.05; **p < .01; *** *p* <.001

To examine the differential contributions, we conducted regression analyses predicting group-level concealment from each type of sensitivity. For completeness, we conducted an identical analysis for distress. In these models, both types of sensitivities were entered simultaneously to control one another. We report bivariate and partial associations of each sensitivity with outcomes in Table 5 (predictors in rows; outcome variables in columns). It should be noted that predicting group-level concealment from Level 1 variables poses a problem, with no best practices to conduct these analyses (Croon & van Veldhoven, 2007). Thus, for this particular subset of analyses, we aggregated the sensitivities to be Level 2 predictors and used the general linear model. This accommodation was not needed for psychological distress, and we examined these relationships with mixed regression models, specifying the identity group as a random intercept. Analyses were conducted in R using *lm* package for general linear models and the *lme4* and *lmerTest* packages for the mixed regression models.

**Supplemental Table 5**.

*Discriminant Validity*

|  |  |  |
| --- | --- | --- |
|  | Group-Level | Psychological |
|  | Concealment | Distress |
| Rejection Sensitivity |  |  |
| β | .86*** | .33*** |
| β (Stigmatization) | .84*** | .30*** |
|  |  |  |
| Stigmatization Sensitivity |  |  |
| β | .54*** | .23*** |
| β (Rejection) | .03 | .05 |

Note: Model controlled for variable in parentheses.

; *** *p* <.001

When controlling for the other type of sensitivity, only rejection sensitivity was associated with group-level concealment. That is, the identity group, in particular, appears to play an important role in facilitating rejection, but not stigmatization, sensitivity.

Finally, we examined a parallel mediation model. We examined the relative indirect contributions of each type of sensitivity in the association of group-level concealment and distress. We conducted this exploratory analysis in PROCESS macro of SPSS using Model 4. Analyses controlled for group-mean-centered concealment. For brevity, we report results graphically in Figure 2. As can be seen, the association of group-level concealment behavior and psychological distress was mediated through rejection sensitivity, *ab* = .08, Boot *SE* = .03, 95% CI [.04, .14], but not stigmatization sensitivity, *ab* = .02, Boot *SE* = .01, 95% CI [-.004, .05].

**Supplemental Figure 1.**

Identity-Based

Rejection Sensitivity

Psychological

Distress

Concealment

Behavior

Group

Individual

Stigmatization

Sensitivity

.23*** (.33***)

.21***

.36***

.23**

.10

Note. *p <.05; **p < .01; *** *p* <.001
